# Identification of a New m6A Regulator-Related Methylation Signature for Predicting the Prognosis and Immune Microenvironment of Patients with Pancreatic Cancer

**DOI:** 10.1155/2023/5565054

**Published:** 2023-05-02

**Authors:** Tianle Zou, Dan Shi, Weiwei Wang, Guoyong Chen, Xianbin Zhang, Yu Tian, Peng Gong

**Affiliations:** ^1^Department of General Surgery and Integrated Chinese and Western Medicine, Institute of Precision Diagnosis and Treatment of Gastrointestinal Tumors, Carson International Cancer Center, Shenzhen University General Hospital, Shenzhen University, Shenzhen, Guangdong 518060, China; ^2^College of Nursing, Shenzhen University Medical School, Shenzhen University, Shenzhen, Guangdong 518060, China; ^3^Hepatobiliary Surgery, People's Hospital of Zhengzhou University and Henan Provincial People's Hospital, Zhengzhou, Henan, China; ^4^School of Public Health, Benedictine University, Lisle, USA

## Abstract

Pancreatic cancer (PC) is a malignant tumor of the digestive system that has a bad prognosis. N6-methyladenosine (m6A) is involved in a wide variety of biological activities due to the fact that it is the most common form of mRNA modification in mammals. Numerous research has accumulated evidence suggesting that a malfunction in the regulation of m6A RNA modification is associated with various illnesses, including cancers. However, its implications in PC remain poorly characterized. The methylation data, level 3 RNA sequencing data, and clinical information of PC patients were all retrieved from the TCGA datasets. Genes associated with m6A RNA methylation were compiled from the existing body of research and made available for download from the m6Avar database. The LASSO Cox regression method was used to construct a 4-gene methylation signature, which was then used to classify all PC patients included in the TCGA dataset into either a low- or high-risk group. In this study, based on the set criteria of |cor| > 0.4 and *p* value < 0.05. A total of 3507 gene methylation were identified to be regulated by m6A regulators. Based on the univariate Cox regression analysis and identified 3507 gene methylation, 858 gene methylation was significantly associated with the patient's prognosis. The multivariate Cox regression analysis identified four gene methylation (PCSK6, HSP90AA1, TPM3, and TTLL6) to construct a prognosis model. Survival assays indicated that the patients in the high-risk group tend to have a worse prognosis. ROC curves showed that our prognosis signature had a good prediction ability on patient survival. Immune assays suggested a different immune infiltration pattern in patients with high- and low-risk scores. Moreover, we found that two immune-related genes, CTLA4 and TIGIT, were downregulated in high-risk patients. We generated a unique methylation signature that is related to m6A regulators and is capable of accurately predicting the prognosis for patients with PC. The findings might prove useful for therapeutic customization and the process of making medical decisions.

## 1. Introduction

Pancreatic cancer (PC) is among the deadliest malignancies, with a mortality rate that ranks among the top four worldwide [1]. At the moment, less than 10% of patients with PC are diagnosed in the early stage of the disease [2, 3]. Due to the fact that most patients are detected at a later stage, they are unable to undergo surgical therapy because this treatment option is not available [4, 5]. The high death rate is mostly attributable to a number of factors, including, but not limited to, the medical history of the family, genetics, the intake of cigarettes, and chronic pancreatitis [6]. PC has continued to have a poor clinical prognosis due to its late presentation with vague symptoms and its early metastatic tendency, despite the breakthroughs in cancer treatments that have occurred during the past few decades [7, 8]. When compared to all other types of solid tumors, the probability of surviving PC for five years is the lowest, at 8% [9, 10]. Therefore, in order to better the prognosis for patients with PC, there is an urgent need to discover new biomarkers for early diagnosis and prospective therapeutic strategies to combat the progression of cancer.

Previous research has demonstrated that mutated genes are the primary cause of cancerous growths. Epigenetic modifications like DNA methylation, histone acetylation, and RNA modification have all been proven to play a role in the development and progression of tumors [11, 12]. These epigenetic modifications have been recognized as new treatment and prognostic targets as a result of the expansion of research into the subject. To this day, researchers have discovered an increasing number of posttranscriptional changes of RNA. It was not until the 1970s that researchers discovered N6-methyladenosine, also known as m6A, which is now thought to be the most common and prolific posttranscriptional modification found in eukaryotic mRNA [13, 14]. Although just 0.1-0.4% of all adenosine in mammals is methylated as a result of m6A RNA, this type of RNA is responsible for around 50% of all methylation ribonucleotides. The alteration of m6A is involved in virtually every stage of the RNA metabolic process, from splicing to decay [15, 16]. There is a growing body of research that acknowledges the significant role that m6A alteration plays in the progression of a variety of disorders, including hypertension, cardiovascular diseases, and acute myeloid leukemia, among others [17, 18]. Emerging research suggests that m6A regulators may be able to mediate gene expression levels in a variety of biological processes, such as the formation, progression, invasion, and metastasis of cancer, and may also be able to function as prognostic indicators [19–22]. In addition, a study demonstrated that there are four distinct types of RNA modification writers, each of which may play an important part in the tumor microenvironment (TME), targeted therapy, and immunotherapy in PC [23, 24]. However, it is not yet known how important the m6A-related genes are in PC from a functional standpoint.

Gene expression profiles have been utilized as a means of locating prognostic genes as novel biomarkers for many types of cancer since the emergence of genome sequencing and screening tools [25, 26]. Several research over the past several years have established a variety of predictive models based on m6A-related genes, m6A-related lncRNAs, and m6A-related eRNAs [27, 28]. RNA methylation is an important epigenetic modification that is involved in the regulation of gene expression in a variety of biological processes [29, 30]. This regulation takes place without any alterations to the fundamental nucleotide sequence. In carcinogenesis, aberrant RNA methylation takes place, and numerous methylation biomarkers have been exploited to predict the prognosis of patients with PC [31, 32]. RNA methylation profiles can be used to provide an accurate prediction as well as suggest potential treatments for cancers. Therefore, research into the predictive significance of m6A-related epigenetic characteristics such as DNA methylation in PC is required.

## 2. Methods

### 2.1. Data Preparation

The level 3 RNA sequencing data, methylation data, and clinical information of pancreatic cancer patients were downloaded from TCGA datasets (TCGA-PAAD, https://portal.gdc.cancer.gov/). m6A RNA methylation-related genes were collected from the known literature and were downloaded from the m6Avar database (http://m6avar.renlab.org/). The m6Avar database was a collection of information pertaining to functional variants that were involved in the m6A alteration process. For the purpose of measuring the DNA methylation data, an Illumina Human Methylation 450 Beadchip (450 K array), was utilized. Across the entirety of the genome, a total of 482,421 CpG sites are going to be analyzed. The association of mean methylation and expression of specific genes in pancreatic cancer was compared via MEXPRESS (https://mexpress.be/).

### 2.2. Identification of m6A Regulator-Related Methylation

To identify methylation regulated by m6A regulators, Pearson's test was performed to examine the correlation between gene methylation value and m6A regulator expression. Pearson's *R* > 0.3 was considered to be statistically significant.

### 2.3. Differentially Expressed Gene (DEG) Analysis

DEG analysis was performed based on the limma package in R software with the set standards.

### 2.4. Gene Set Enrichment Analysis (GSEA)

We tested for the overrepresentation of differentially methylated genes or genes linked with differential methylation risk scores by using gene sets from the Molecular Signatures Database version 6.2 (MSigDB). The reference gene sets were Hallmark, Gene Ontology (GO), and Kyoto Encyclopedia of Genes and Genomes (KEGG). GSEA was carried out with the help of the fgsea package (version 1.4.1), and 10,000 permutations were used in order to locate enriched pathways that were shared by the high-risk group and the low-risk group. |NES| values greater than one and a false discovery rate of less than 0.05 percent were regarded as statistically significant.

### 2.5. Prognosis Model Construction

Firstly, for the input gene methylation data, univariate assays were utilized to identify the gene methylation tightly correlated with patient survival. Then, LASSO Cox regression of overall survival (OS) was carried out to identify survival-related gene methylation. Multivariate assays were used for prognosis model construction (Risk score = Methylation level of gene *A* × coef *A* + Methylation level of gene *B* × coef *B* + ⋯+Methylation level of gene *N* × coef *N*), and the risk score of each sample in all the datasets was calculated based on the signature. For survival analysis, the samples were divided into a high-risk group and a low-risk group based on the median cutoff value of the risk score. Kaplan-Meier (KM) and receiver operating characteristic (ROC) curves were used to explore the prognostic significance of the prognosis signature.

### 2.6. Immune-Related Analysis

Comparisons were made between the CIBERSORT, ESTIMATE, MCPcounter, EPIC, Xcell, and TIMER algorithms in order to evaluate the differences in cellular components or cellular immune responses between the high-risk group and the low-risk group based on the prognostic signature [33–36]. A heatmap was used to uncover the changes in the immune response that occurred under the influence of several algorithms. In addition, the potential response of patients to immunotherapy was inferred by the tumor immune dysfunction and exclusion (TIDE) score. Generally, a lower TIDE score indicates a better response to immunotherapy, in which the patients with TIDE score < 0 were regarded as immunotherapy responders, otherwise, nonresponders. For the purpose of quantifying the differences in tumor-infiltrating immune cell subgroups between the two groups, the single sample gene set enrichment analysis (ssGSEA) algorithm was utilized.

### 2.7. Statistical Analysis

Data were analyzed using Bioconductor packages in R software(version 4.0.2, R Core Team, Massachusetts, USA). The differences between clinical tissues were tested by Student's *t*-test. Log-rank test and Kaplan-Meier analysis were used to compare the OS between groups. The Cox proportional hazards model was used to examine the independent significance of relevant clinical factors. A *p* < 0.05 was considered statistically significant.

## 3. Results

### 3.1. Identification of m6A Regulators in PC

**Figure 1:**
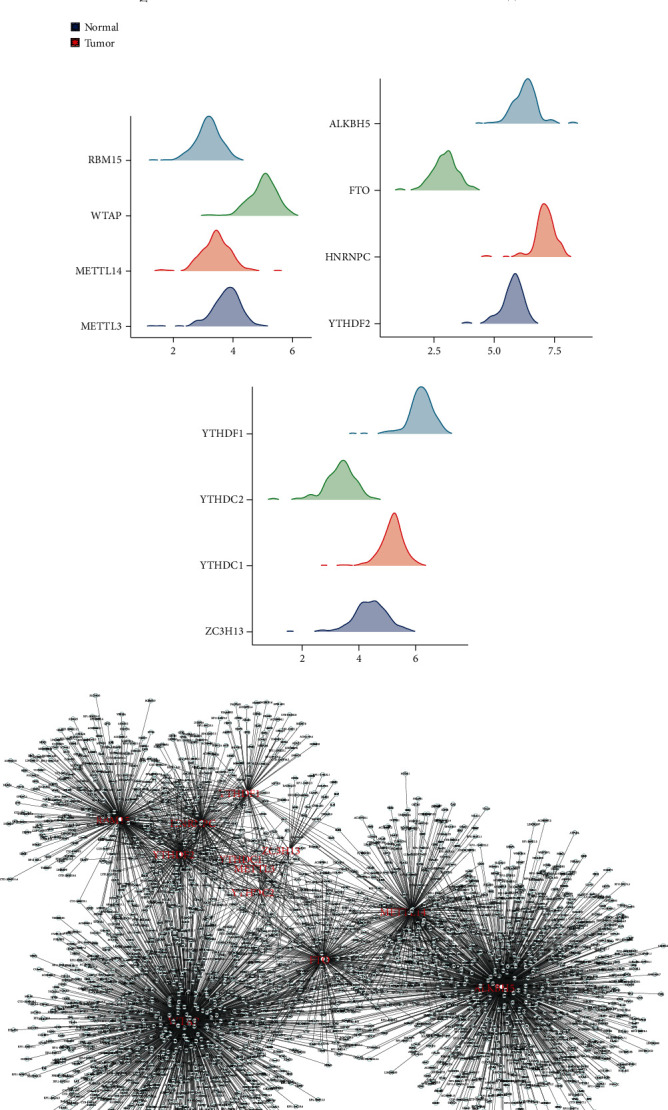
Identification of the gene methylation regulated by m6A regulators. (a) The expression level of the m6A regulator in pancreatic cancer and normal tissue; (b–d) the expression level of m6A regulators in TCGA-PAAD; (e) the gene methylation regulated by the m6A regulators. ^∗∗∗^*p* < 0.001, ^∗∗^*p* < 0.01, ^∗^*p* < 0.05.

It has been confirmed that the dysregulation of m6A methylation was involved in the progression of various tumors. Thus, our group extracted the expressions of identified m6A regulator, including METTL3, METTL14, WTAP, RBM15, ZC3H13, ALKBH5, FTO, HNRNPC, YTHDF2, YTHDF1, YTHDC2, and YTHDC1. The result indicated that all these m6A regulators showed an aberrant expression pattern in pancreatic cancer (Figure 1(a)). The expression distribution of all these m6A regulators was shown in Figures 1(b)–1(d). Based on the set criteria of |cor| > 0.4 and *p* value < 0.05. A total of 3507 gene methylation were identified to be regulated by m6A regulators (Figure 1(e)).

### 3.2. Prognosis Model Construction

**Figure 2:**
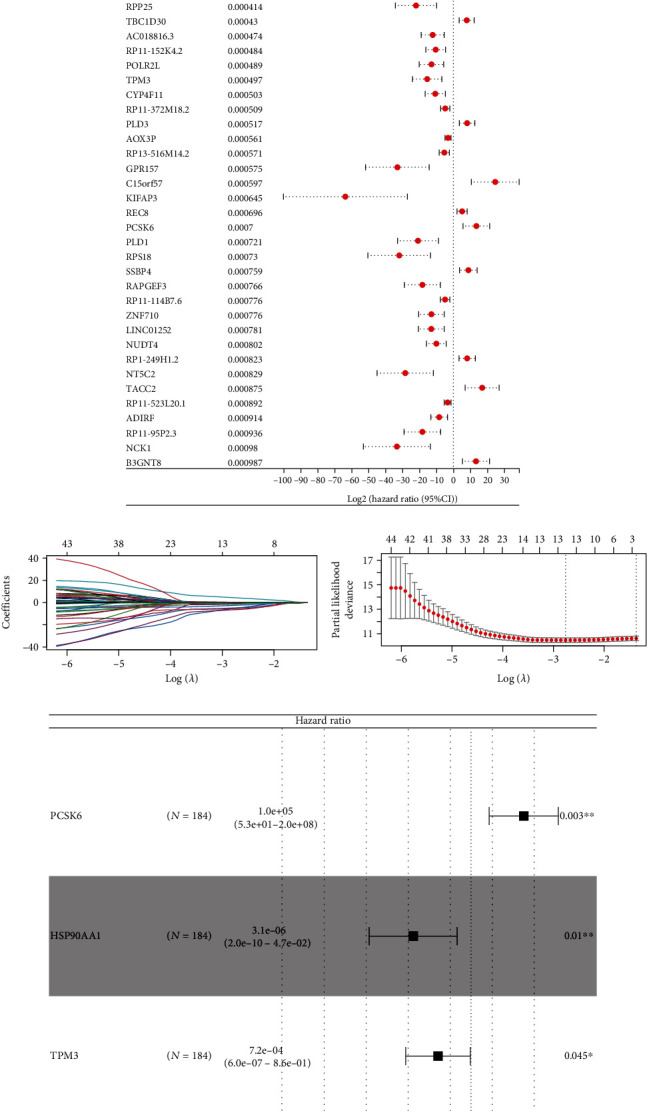
Screening of prognosis-related gene methylation. (a) The top 50 gene methylation tightly correlated with patients' prognosis; (b, c) LASSO regression analysis; (d) multivariate Cox regression analysis. ^∗∗^*p* < 0.01, ^∗^*p* < 0.05.

**Figure 3:**
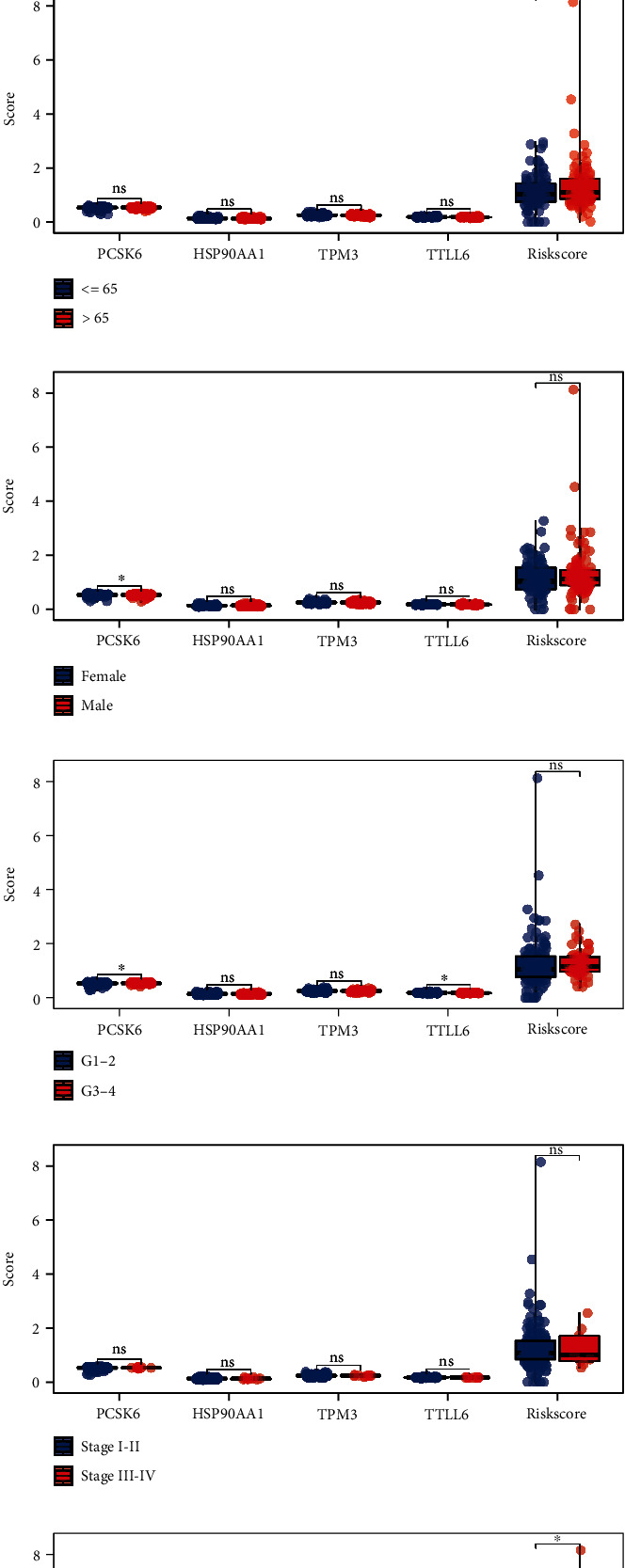
Prognosis signature. (a) The overview of the prognosis model; (b) KM survival curve of high- and low-risk patients; (c–e) the ROC curve of 1-, 3-, and 5-year survival; (f–k) clinical correlation of model gene methylation and risk score.

Based on the univariate assays and identified 3507 gene methylation, 858 gene methylation was distinctly related to the clinical outcome of PC patients. Among which, the top 50 prognosis-related gene methylations were selected for visualization and further analysis (Figure 2(a)). LASSO regression algorithm was used for data dimension reduction (Figures 2(b) and 2(c)). Finally, the multivariate assays identified four gene methylation to construct a prognosis model with the formula of Risk score = Methylation level of PCSK6 × 11.54 + Methylation level of HSP90AA1 × −12.68 + Methylation level of TPM3 × −7.24 + Methylation level of TTLL6×−17.35 (Figure 2(d)). The overview of our prognosis signature was shown in Figure 3(a), in which a higher percentage of dead cases was observed in the high-risk group. Survival assays indicated that the patients in the high-risk group tend to have a worse prognosis (Figure 3(b), HR = 2.82, *p* < 0.001). ROC curves showed that our prognosis signature had a good prediction ability on patient survival (Figures 3(c)–3(e)) (1-year AUC = 0.68, 3-year AUC = 0.809, and 5-year AUC = 0.806).

### 3.3. Clinical Correlation Analysis

**Figure 4:**
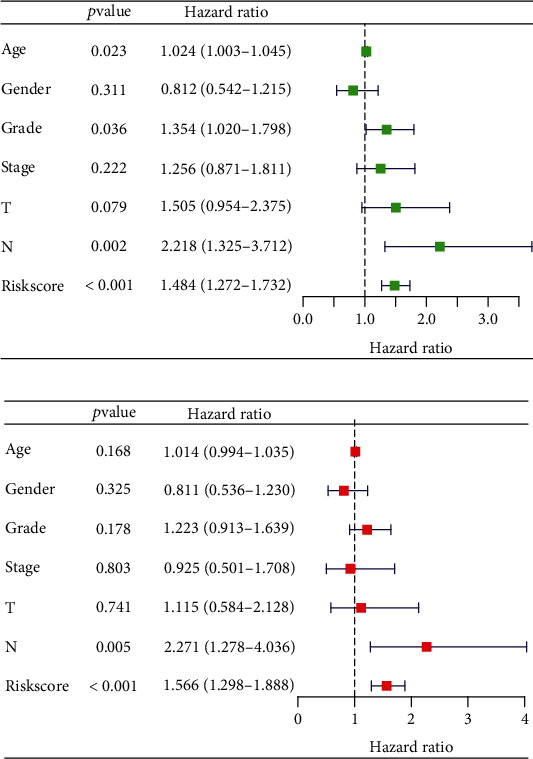
Prognostic factors for overall survival by univariate (a) and multivariate (b) analysis.

To better understand the prognosis differences between high- and low-risk patients, we then performed a clinical correlation analysis. Results indicated that no significant differences were observed in patients with different ages (Figure 3(f)); PCSK6 was upregulated in female patients (Figure 3(g)); PCSK6 was overexpressed in G1-2 patients (Figure 3(h)); no significant differences were observed in patients with different clinical stages (Figure 3(i)); the T3-4 patients tend to have a lower HSP90AA1, while a higher risk score level compared to the T1-2 patients (Figure 3(j)); no significant differences were observed in patients with different N stages (Figure 3(k)). Finally, we evaluated the roles of the novel model and other clinicopathologic parameters on the prognosis of PC with univariate and multivariate assays. As shown in Figures 4(a) and 4(b), we confirmed that the novel prognostic model was an independent prognostic factor for overall survival in PC patients.

### 3.4. Biological Enrichment Analysis

**Figure 5:**
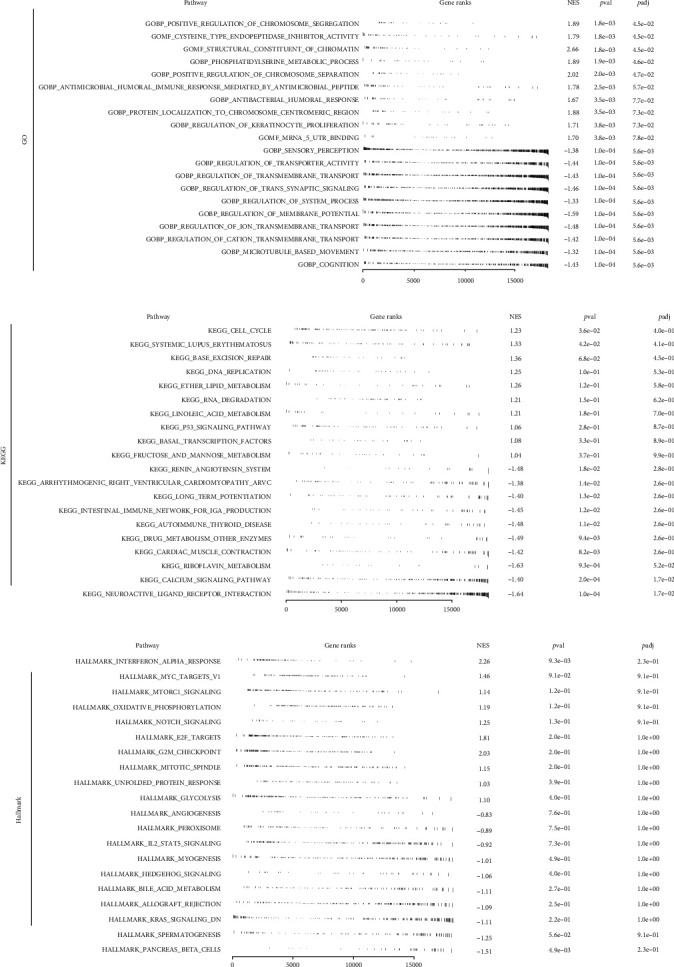
Biological enrichment analysis. (a) GSEA analysis based on the GO gene set; (b) GSEA analysis based on the KEGG gene set; (c) GSEA analysis based on the Hallmark gene set.

Underlying biological pathway difference can lead to different prognosis performance. For the GSEA analysis based on GO, the terms positive regulation of chromosome segregation, cysteine-type endopeptidase inhibitor activity, structural constituent of chromatin, phosphatidylserine metabolic process, and positive regulation of chromosome separation were the top five enriched terms (Figure 5(a)). For the GSEA analysis based on KEGG analysis, the cell cycle, systemic lupus erythematosus, base excision repair, DNA replication, and ether lipid metabolism were the top five enriched terms (Figure 5(b)). For the GSEA analysis based on the Hallmark gene set, the terms interferon alpha response, MYC targets, mTORC1 signaling, oxidative phosphorylation, and Notch signaling were the top five enriched terms (Figure 5(c)).

### 3.5. Immune-Related Analysis

**Figure 6:**
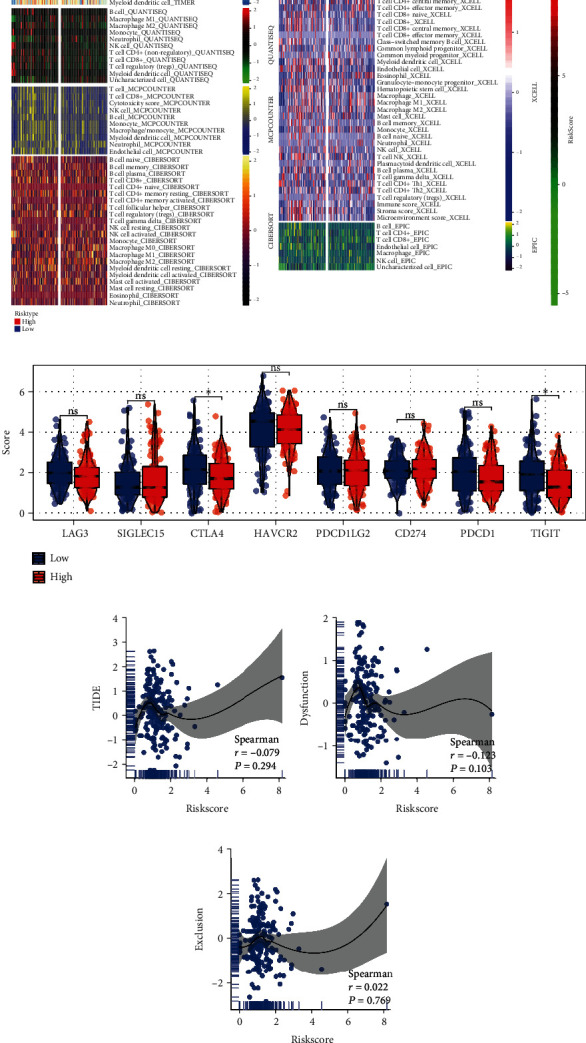
Immune-related analysis. (a) Immune infiltration analysis based on multiple algorithms; (b) several immune checkpoint genes in high- and low-risk groups; (c–e) correlation of risk score with TIDE, dysfunction, and exclusion. ^∗^*p* < 0.05.

The tumor immune microenvironment plays an important role in tumor progression. We next quantified the tumor immune microenvironment based on multiple algorithms, including CIBERSORT, ESTIMATE, MCPcounter, EPIC, Xcell, and TIMER. The result indicated a different immune infiltration pattern in patients with high- and low-risk scores (Figure 6(a)). Moreover, we found that two immune-related genes CTLA4 and TIGIT were downregulated in high-risk patients (Figure 6(b)). Also, we explored the underlying effect of risk score on TIDE, immune dysfunction, and immune exclusion, while no significant difference was found (Figures 6(c)–6(e)).

## 4. Discussion

PC is one of the most dangerous types of malignant tumors [37]. According to the latest statistics on cancer in 2019, the incidence and mortality rates of pancreatic cancer are only second to those of colorectal cancer among malignancies that affect the digestive tract [38, 39]. Studies conducted in clinical settings have indicated that resistance to chemotherapy is the single most important factor that restricts treatment options for pancreatic cancer. This factor also adds to the disease's low survival rate and bad prognosis [40, 41]. The TNM staging system is typically applied in practice for the purposes of classifying cancer patients and choosing appropriate treatments for them [42]. Yet, due to the wide variety of cancers, even those at the same stage may respond differently to therapy. High-throughput sequencing has grown increasingly prevalent in cancer diagnosis and treatment in recent years. In addition, there has been a significant number of research conducted on the process by which RNA is altered in cancer. The various m6A signatures have been identified as predictive prognosis models in many cancers, such as hepatocellular carcinoma, renal cell carcinoma, lung adenocarcinoma, breast cancer, and glioma [43–45].

DNA methylation, as a major epigenetic alteration, has been implicated in the regulation of gene expression by DNA methyltransferase (DNMT) [46, 47]. In addition, the importance of DNA methylation in the development and progression of cancers has been established beyond a reasonable doubt. The prognosis of patients with PC has been predicted using a variety of methylation indicators. In PC, the prognostic prediction model that was based on the DNA methylation site demonstrated greater prediction effectiveness. In a previous study, an unsupervised consistent clustering approach was used to identify two PAAD methylation subtypes, which were dubbed Cluster1 and Cluster2. Cluster2 was shown to be linked with a more favorable prognosis than Cluster1, which was found to be more common. Fourteen methylation genes that are exclusive to each PAAD subtype were found, and these genes might be used as molecular markers to describe the different methylation patterns that are associated with the two PAAD subtypes [48]. However, the DNA methylation signature of m6A regulators has not been investigated in the prognostic prediction of PC. In this study, based on the set criteria of |cor| > 0.4 and *p* value < 0.05. A total of 3507 gene methylation were identified to be regulated by m6A regulators. The LASSO regression algorithm was used for data dimension reduction. Finally, the multivariate Cox regression analysis identified four gene methylation(PCSK6, HSP90AA1, TPM3, and TTLL6) to construct a prognosis model. Survival analysis indicated that the patients in the high-risk group tend to have a worse prognosis. ROC curves showed that our prognosis signature had a good prediction ability on patients' survival. Our findings highlighted the potential of the novel model used as a novel prognostic biomarker for PC patients.

Immunotherapy has only very recently been recognized as a potential new treatment for PC [49]. The extracellular matrix (ECM), stromal cells, tumor vasculature, and numerous immune system cells all contribute to the TME, which is what encourages the development and progression of cancer [50, 51]. It is common knowledge that immune-suppressing cells might play a role in the development of immune evasion in the TME, which in turn helps tumor spread and progression. Tregs are a well-known kind of immunosuppressive cells, and it has been demonstrated that their number is connected with the prognosis of patients [52, 53]. This suggested that the number of Tregs may be an efficient marker for determining the clinical outcome of patients with PC. Immune suppression is one of the most recognizable symptoms of PC, which is caused by the oncogenic drivers. Because of the metabolic reprogramming of tumor cells, which allows them to facilitate the aerobic glycolysis process in order to adapt to their heterogeneous microenvironment, the majority of solid tumors depend heavily on aerobic glycolysis as a source of energy production [54]. TME consists of more than just the tumor cells themselves; it also contains the immune cells, fibroblasts, and fibroblasts that surround the tumor [55]. PC cells are difficult to penetrate and exist in a low-perfusion environment, both of which favor metabolic rearrangement in the PC [56]. This is because the PC is composed of dense connective tissue and has a vascular milieu. Then, we found a different immune infiltration pattern in patients with high- and low-risk scores. In addition, we discovered that macrophage M0 cells were significantly different between high-risk and low-risk signatures. This suggests that macrophage M0 cells might be directly associated to the signature; however, the mechanism behind this relationship has to be researched in more depth. Thus, we came to the conclusion that the tumor immunosuppressive microenvironment might be to blame for the dismal prognosis that high-risk PC patients experience.

In addition, the expression and control of immune checkpoint molecules (such as PD-1, PD-L1, PD-L2, and CTLA-4) also play a vital role in the regulation of the immune response [57]. This is accomplished by inhibiting the activation of protective immune cells and enhancing immune surveillance [58]. Thus, it is not difficult to comprehend why the expression of immune checkpoint molecules was found to be higher in the high-risk group in our study. Immune checkpoint drugs are typically more effective in cases with higher expression of immune checkpoint molecules (ICIs) [59, 60]. In this study, we found two immune-related genes CTLA4 and TIGIT were downregulated in high-risk patients. The results need to be further studied. I suggested that the function of CTLA4 and TIGIT in advanced PC may be different from patients with early stage.

Several limitations exist in this study. Firstly, the clinical data that was obtained from the TCGA databases was scant and lacked essential details. Secondly, this was a retrospective study, and therefore, it lacked novel clinical samples and data.

## 5. Conclusion

We generated a unique methylation signature that is related to m6A regulators and is capable of accurately predicting patients' prognoses when they have PC. This model can be used to aid doctors in the selection of the therapy that is most appropriate for different individuals, and it can, thus, optimize the clinical outcome for patients' PC.

## Data Availability

The datasets used and/or analyzed during the current study are available from the corresponding author on reasonable request.
